# microRNA-181b is increased in cystic fibrosis cells and impairs lipoxin A_4_ receptor-dependent mechanisms of inflammation resolution and antimicrobial defense

**DOI:** 10.1038/s41598-017-14055-y

**Published:** 2017-10-18

**Authors:** Anna Maria Pierdomenico, Sara Patruno, Marilina Codagnone, Felice Simiele, Veronica Cecilia Mari, Roberto Plebani, Antonio Recchiuti, Mario Romano

**Affiliations:** 1 Department of Medicine and Aging Sciences, “G. D’Annunzio” University of Chieti-Pescara, 66013 Chieti, Italy; 2 Department of Medical, Oral, and Technological Sciences, “G. D’Annunzio” University of Chieti-Pescara, 66013 Chieti, Italy; 3 Center on Aging Science and Translational Medicine (CeSI-MeT) “G. D’Annunzio” University of Chieti-Pescara, 66013 Chieti, Italy

## Abstract

The involvement of microRNA (miR) in cystic fibrosis (CF) pathobiology is rapidly emerging. We previously documented that miR-181b controls the expression of the ALX/FPR2 receptor, which is recognized by the endogenous proresolution ligand, lipoxin (LX)A_4_. Here, we examined whether the miR-181b-ALX/FPR2 circuit was altered in CF. We examined human airways epithelial cells, normal (16HBE14o-), carrying the ΔF508 mutation (CFBE41o-) or corrected for this mutation (CFBE41o-/CEP-CFTR wt 6.2 kb), as well as monocyte-derived macrophages (MΦs) from CF patients. CFBE41o- cells exhibited higher miR-181b and reduced ALX/FPR2 levels compared to 16HBE14o- and CFBE41o-/CEP-CFTR wt 6.2 kb cells. An anti-mir-181b significantly enhanced ALX/FPR2 expression (+ 60%) as well as LXA_4_-induced increase in transepithelial electric resistance (+ 25%) in CFBE41o- cells. MΦs from CF patients also displayed increased miR-181b (+ 100%) and lower ALX/FPR2 levels (− 20%) compared to healthy cells. An anti-mir-181b enhanced ALX/FPR2 expression (+ 40%) and normalized receptor-dependent LXA_4_-induced phagocytosis of fluorescent-labeled zymosan particles as well as of *Pseudomonas aeruginosa* by CF-MΦs. These results provide the first evidence that miR-181b is overexpressed in CF cells, impairing some mechanisms of the ALX/FPR2-dependent pathway of inflammation resolution. Thus, targeting miR-181b may represent a strategy to enhance anti-inflammatory and anti-microbial defense mechanisms in CF.

## Introduction

Non resolving lung inflammation is the main cause of disability and death in patients with cystic fibrosis (CF), the most common autosomal recessive genetic disease^1^. Mutations in the gene encoding the CF transmembrane conductance regulator (CFTR), a regulatory protein of ion transport expressed in a broad variety of cells and tissues, are the cause of CF^2,3^. Although the respiratory and the intestinal districts are primarily affected, CF is a systemic disease in which nearly every cell type, including blood cells^4–6^, and tissue is dysregulated as consequence of CFTR mutations^7^. Inflammation in CF begins early in life even before infections^8^, can be exacerbated by bacterial colonization promoted by the CFTR-loss-of-function-dependent reduction in the periciliary fluid volume that impairs mucociliary clearance^9^, and is exaggerated relatively to the bacterial burden^8^. Given the high incidence of inflammation-related disabilities and life losses among patients, understanding why inflammation fails to resolve in CF is overtly important for structuring better therapeutic approaches.

The concept that the resolution phase of the inflammatory response is an active process governed by an array of peptide and lipid mediators and their cognate receptors is supported by numerous observations^10^. Small lipid mediators, generated *in vivo* during inflammation resolution, are derived from polyunsaturated fatty acids, i.e arachidonic, eicosapentaenoic, docosahexaenoic acid, and comprehensively termed specialized proresolving lipid mediators. These, include lipoxins (LX), resolvins (Rv), protectins and maresins^11^, which are generated by the catalytic activity of lipoxygenases (LO) and exert regulatory and counterregulatory functions on key processes of the inflammatory response to promote resolution and tissue repair^11^.

LXA_4_ and RvD1 activate a G protein-coupled receptor termed ALX/FPR2^12,13^. This proresolving receptor is also recognized by the endogenous anti-inflammatory peptide, Annexin A1^14^. *In vivo*, myeloid-targeted overexpression of human ALX/FPR2 in mice reduced PMN infiltration during acute peritonitis and a left-shifted the dose response to its ligands^15^, whereas ALX/FPR2 KO mice manifested an impaired resolution phenotype^16^. Consistent with this, ALX/FPR2 and its proresolution agonists are defective in human diseases characterized by non resolving inflammation, such as asthma, obesity and atherosclerosis^17–20^. Thus, mechanisms that control ALX/FPR2 expression may represent relevant targets to potentiate endogenous anti-inflammatory pathways.

Evidence indicates that endogenous mechanisms that normally limit the severity and duration of inflammation, promoting its timely resolution, are defective in CF patients. For instance, LXA_4_ levels are significantly decreased in CF pediatric patients^21^. Also, AnxA1 is downregulated in nasal cells from CF patients^22^, and platelets from CF patients have an impaired LX-biosynthesis capability^4^. Finally, a recent study indicates that *Pseudomonas aeruginosa*, one of the main opportunistic pathogens colonizing CF airways, impairs the transcellular biosynthesis of 15-epi-LXA_4_ by secreting a soluble CFTR inhibitory factor^23^. Of interest, in preclinical models of CF, the ALX/FPR2 agonists LXA_4_, AnxA1, RvD1, and 15-epi-LXA_4_ proved beneficial in dampening neutrophil infiltration, reducing the release of pro-inflammatory mediators by CF cells, and limiting collateral tissue damage, demonstrating their therapeutic potential for treating chronic lung inflammation^21,23–25^. Hence, uncovering the molecular mechanisms by which ALX/FPR2 is regulated in CF can offer the basis for a better understanding of CF pathophysiology and the designing of innovative therapeutics that activate endogenous resolution circuits.

We recently uncovered genetic and epigenetic mechanisms that regulate ALX/FPR2 expression^26,27^, including its inhibition by microRNA (miR)-181b^26^, which also blunts receptor-dependent anti-microbial and anti-inflammatory cellular mechanisms^26^. Therefore, we asked whether the ALX/FPR2- LXA_4_ circuit and its regulation by miR-181b was altered in CF.

Herein, we provide evidence that miR-181b is overexpressed in CF cells, impairing the ALX/FPR2-dependent pathway of inflammation resolution. Thus, targeting miR-181b may represent a novel strategy to enhance anti-inflammatory and anti-microbial defense mechanisms in CF.

## Results

### ALX/FPR2 expression is downregulated in CF airways cells: involvement of transcriptional and epigenetic events

**Figure 1:**
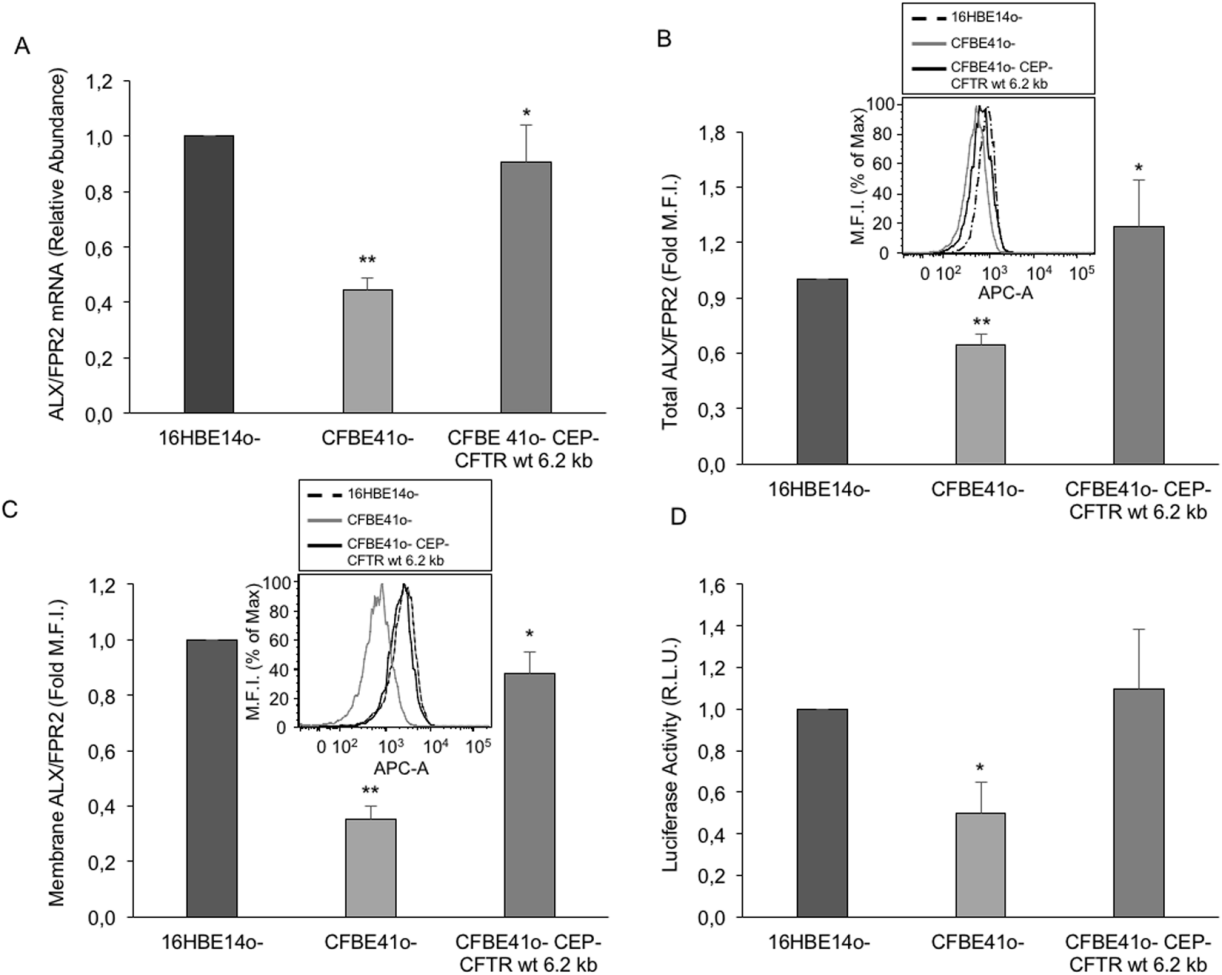
ALX/FPR2 expression in normal and CF human respiratory epithelial cells. ALX/FPR2 expression was evaluated in 3 human respiratory epithelial cell lines: normal (16HBE14o-), CF (CFBE41o-) and CF normalized by overexpression of wild type CFTR (CFBE41o- CEP-CFTR wt 62 kb). (**A**) ALX/FPR2 was evaluated by real-time PCR as reported in material and methods. Results are mean ± SEM from n = 3. *p = 0.03 vs CFBE41o-; **p = 0.002 vs 16HBE14o-. (**B**) Total ALX/FPR2 protein was assessed by flow cytometry in permeabilized cells. Bars depict mean ± SEM from n = 3. *p < 0.05 vs CFBE41o-; ***p = 0.0008 vs 16HBE14o-. The inset shows a representative histogram. (**C**) Membrane ALX/FPR2 was evaluated in non permeabilized cells as in (b). Data are mean ± SEM from n = 3. **p = 0.04 vs CFBE41o-; ***p = 0.0006 vs 16HBE14o-. The inset shows a representative histogram. (**D**) ALX/FPR2 promoter activity was measured in cells transfected with a pGL4 plasmid containing or not the 346 bp sequence of the ALX/FPR2 core promoter upstream the luciferase reporter gene. Luciferase activity was measured 48 h post-transfection. Values were normalized for protein concentration. Results are mean ± SEM from 3 independent experiments carried out in duplicate. *p = 0.01 vs 16HBE14o-.

We and others reported that LXA_4_ generation is impaired in CF^4,21^. Here, we asked whether the expression of ALX/FPR2, the LXA_4_ receptor, was also altered in CF cells. To this end, we evaluated ALX/FPR2 mRNA and protein expression in 16HBE14o- (normal), CFBE41o- (CF) and CFBE41o-/CEP-CFTR wt 6.2 kb (CF corrected) human respiratory epithelial cells. CFBE41o- cells displayed lower ALX/FPR2 mRNA (~ − 60%) and protein, both total and cell membrane (~ − 40–50%), compared to 16HBE14o- cells (Fig. 1A–C).

To investigate on mechanisms underlying these changes, we analyzed ALX/FPR2 transcriptional activity. CFBE41o-cells, transfected with a plasmid expressing the ALX/FPR2 core promoter upstream the luciferase gene^28^, exhibited significantly lower luciferase activity compared to 16HBE14o- or CFBE41o-/CEP-CFTR wt 6.2 kb cells (Fig. 1D). These results indicate that the ALX/FPR2 transcriptional machinery may be altered in CF cells.

**Figure 2:**
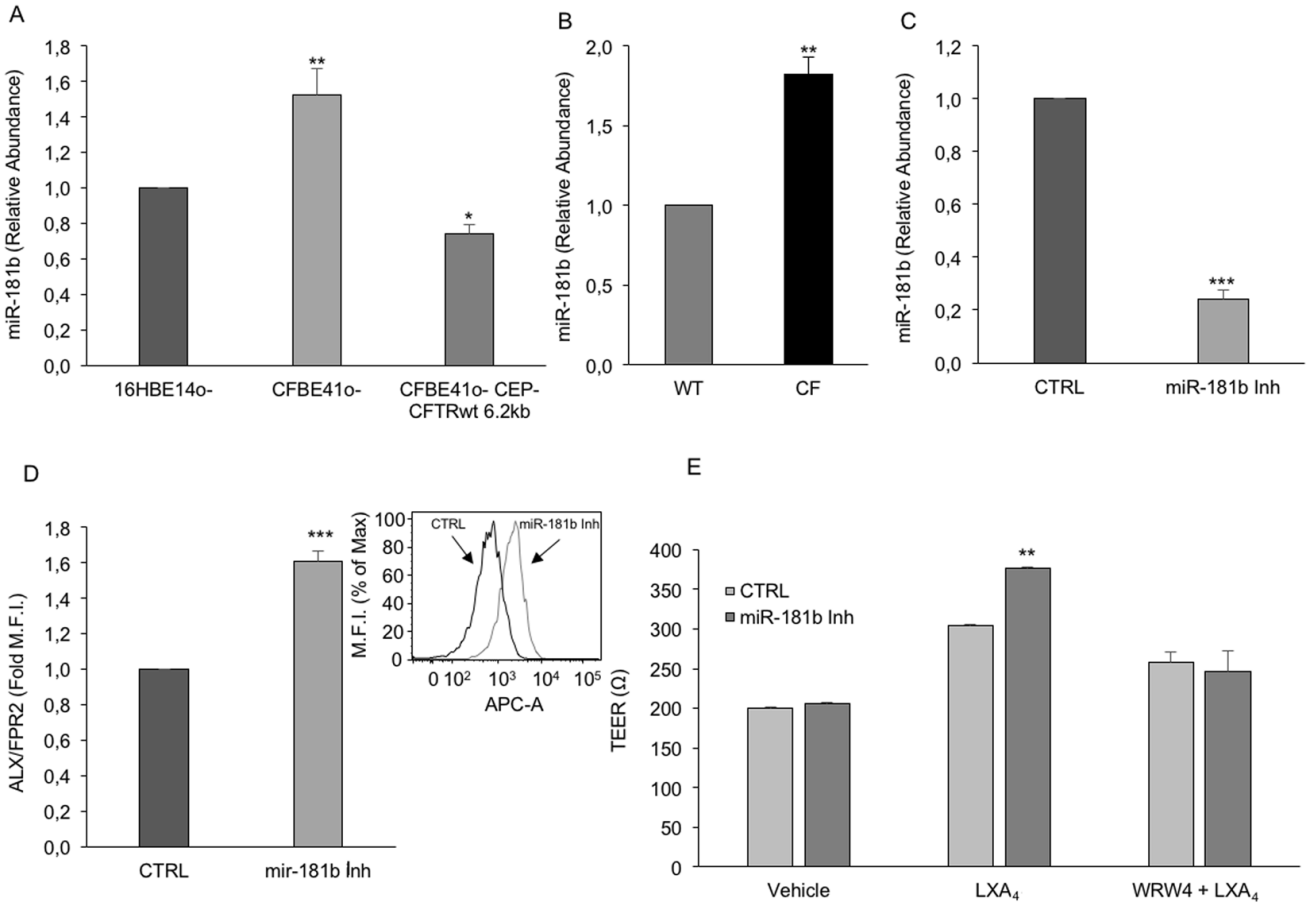
miR-181b is overexpressed in CF airway cells and controls ALX/FPR2 expression and signaling. (**A**) miR-181b levels were evaluated by real-time PCR in normal (16HBE14o-), CF (CFBE41o-) and normalized CF (CFBE41o- CEP-CFTR wt 62 kb) epithelial respiratory cells. RNU6 and SNORD95 were used for normalization. Bars are mean ± SEM from n = 3. *p = 0.04 vs CFBE41o-; **p = 0.02 vs 16HBE14o-. (**B**) miR-181b levels in primary bronchial epithelial cells from 3 non-CF subjects and 3 CF patients carrying the ΔF508/ΔF508 mutation. Bars are mean ± SEM; **p = 0.0027 (**C**) CFBE41o- were transfected with a miR-181b inhibitor. miR-181b expression was evaluated by real-time PCR 24 h post transfection. Results are mean ± SEM from 3 separate transfections. ***p = 0.0001. (**D**) Total ALX/FPR2 protein expression was evaluated by flow cytometry in CFBE41o-cells, transfected with either a negative control or a miR-181b inhibitor. ALX/FPR2 levels were evaluated 48 h post-transfection. Bars depict mean ± SEM from 3 separate transfections. ***p = 0.0005. The inset shows a representative histogram. (**E**) CFBE41o- were transfected with a negative control or a miR-181b inhibitor. Transepithelial electrical resistance (TEER) was measured 48 h post-transfection using a EVOM Voltohmmeter. Results are expressed as mean ± SEM from 3 separate transfections. **p = 0.0026 vs CTRL.

On the other hand, we recently uncovered epigenetic regulatory mechanisms of ALX/FPR2 expression, namely chromatin post-translational modifications^27^ and mir-181b expression^26^. Therefore, we examined whether this miR was involved in ALX/FPR2 expression in CF cells. Figure 2 shows that CFBE41o- cells expressed higher miR-181b levels compared to 16HBE14o- and CFBE41o-/WT cells. We observed a similar increment in bronchial airway primary cells collected from 3 non-CF subjects and 3 CF patients homozygous for the ΔF508 mutation (Fig. 2B).

To analyze the relationship between miR-181b and ALX/FPR2 expression, we downregulated miR-181b using a specific inhibitor (Fig. 2C). miR-181 downregulation enhanced by ~ 60% ALX/FPR2 protein expression in CFBE41o-cells (Fig. 2D), although it did not change ALX/FPR2 mRNA expression (results not shown). To determine whether this increment was associated with enhanced LXA_4_-induced signaling, we evaluated trans epithelial electrical resistance (TEER). LXA_4_ increased TEER by ~ 50% in CFBE41o- cells (Fig. 2E). When miR-181b expression was downregulated, the effect of LXA_4_ was enhanced by ~ 25% (Fig. 2E). This effect was receptor-dependent, since it was abrogated by WRW4, an established ALX/FPR2 antagonist^29^ (Fig. 2E). We also evaluated the impact of miR-181b inhibition on the release of selected cytokines (IL-8, IL-6, RANTES) by CFBE41o- cells, exposed or not to LXA_4_. However, we were unable to detect significant changes in this experimental setting (results not shown).

### miR-181b and ALX/FPR2 expression in CF macrophages

**Figure 3:**
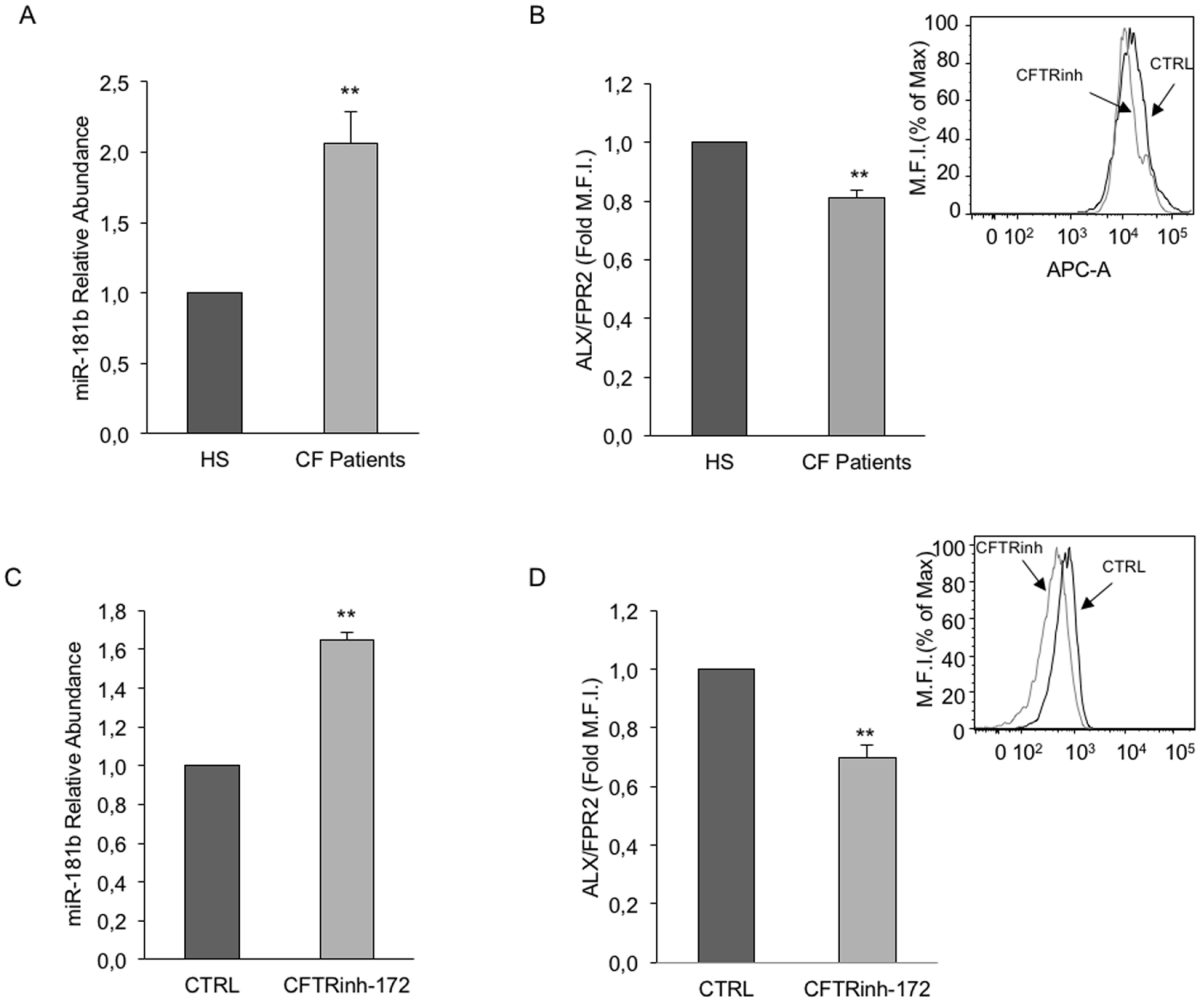
miR-181b and ALX/FPR2 expression in normal and CF-MΦs. MΦs and CF- MΦs were obtained by exposing peripheral blood monocytes from 3 healthy volunteers (HS) and 3 CF patients to GM-CSF for seven days. (**A**) miR-181b levels were determined by real-time PCR. Bars represent mean ± SEM. **p = 0.009. (**B**) ALX/FPR2 expression in MΦs and CF-MΦs were evaluated by flow cytometry. Data are mean ± SEM. **p = 0.0019. The inset shows a representative histogram. (**C**) Normal MΦs were exposed to vehicle (CTRL) or CFTRinh-172 (10 µM) for 30 min. miR-181b levels were determined by real-time PCR. Results are mean ± SEM from n = 3 with duplicates. **p = 0.001. (**D**) Total ALX/FPR2 levels in normal MΦs exposed to vehicle (CTRL) or CFTRinh-172 (10 µM) levels in were quantitated by flow cytometry. Bars represent mean ± SEM from n = 3. **p = 0.0019. The inset shows a representative histogram.

Given the pivotal role of macrophages (MΦs) in inflammation resolution^30^ and the capability of LXA_4_ to stimulate ALX/FPR2-dependent pro-resolving signaling in these cells^31^, we determined miR-181b and ALX/FPR2 levels in MΦs isolated from CF patients and age-matched healthy subjects. CF-MΦs displayed higher miR-181b (~ + 100%, p = 0.009) and lower ALX/FPR2 levels (~ − 20%; p = 0.0019) compared to cells from HS (Fig. 3A and B). Notably, in normal MΦs CFTRinh-172, a selective CFTR inhibitor^29^, for 24 h, enhanced miR-181b (~ + 60%; p = 0.001) and reduced ALX/FPR2 expression (~ − 30%; p = 0.0019), reminiscent of the CF- MΦs profile (Fig. 3C and D). These results indicate that CFTR controls miR-181b expression, which in turn downregulates ALX/FPR2 levels.

### Inhibition of miR-181b upregulates ALX/FPR2 expression and LXA_4_-induced responses in CF- MΦs

**Figure 4:**
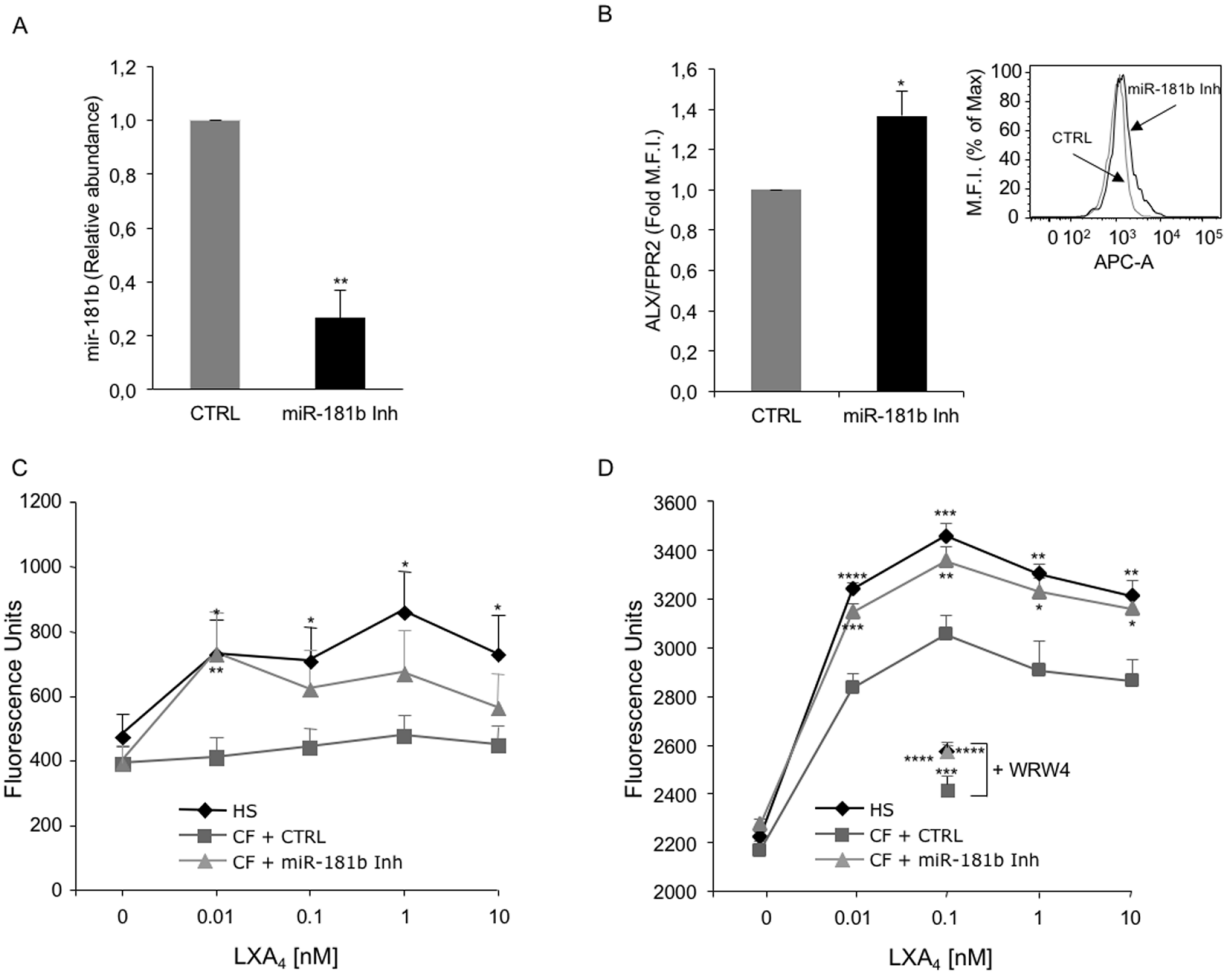
miR-181b inhibition upregulates ALX/FPR2 expression and agonist-induced phagocytosis in CF-MΦs. (**A**) MΦs from healthy subjects were transfected with either a negative control (CTRL) or a miR-181b inhibitor for 24 h miR-181b expression was evaluated by real-time PCR. Bars represent mean ± SEM from 3 independent transfections. **p = 0.0013. (**B**) MΦs were treated as in (A) and total ALX/FPR2 expression was determined by flow cytometry. WRW4 (10 μM) was added to samples incubated with 0.1 nM LXA_4_. Bars are mean ± SEM from 3 independent transfections. *p = 0.019. The inset shows a representative histogram. (**C**) HS or CF-MΦs (5 × 10^5^/well) transfected either with a negative control or with a miR-181b inhibitor, were exposed to the indicated concentrations of LXA_4_. Cells were incubated with FITC-labelled zymosan particles for 30 min at 37 °C and phagocytosis was assessed by measuring fluorescence with a Synergy H1 microplate reader. Data points are mean ± SEM from separate experiments with cells from 4 healthy donors and 4 CF patients. *p = 0.017 (HS vs CF, 0.01 nM LXA_4_); *p = 0.032 (HS vs CF, 0.1 nM LXA_4_); *p = 0.011 (HS vs CF, 1 nM LXA_4_); *p = 0.045 (HS vs CF, 10 nM LXA_4_); **p = 0.003 (CF vs CF + miR-181b inhibitor). (**D**) HS or CF-MΦs (2 × 10^5^/well) were incubated for 1 h at 37 °C with PAO1-GFP at 1:200 cell:bacteria ratio. Phagocytosis of PA01-GFP was determined by measuring total fluorescence (Ex 485 nm/Abs 530 nm) using a plate reader (Synergy, BioTek). Data points are mean ± SEM from separate experiments with cells from 3 healthy donors and 3 CF patients. *p = 0.021 (CF vs CF + miR-181b inhibitor, 1 nM LXA_4_); *p = 0.012 (CF vs CF + miR-181b inhibitor, 10 nM LXA_4_); ** p = 0.009 (CF vs CF + miR-181b inhibitor, 0.1 nM LXA_4_); **p = 0.004 (HS vs CF, 1 and 10 LXA_4_); ***p = 0.0003 (CF vs CF + miR-181b inhibitor, 0.01 nM LXA_4_); ***p = 0.0007 (HS vs CF, 0.1 nM LXA_4_); ***p = 0.00062 (CF vs CF + WRW4, 1 nM LXA_4_); ****p = 0.000006 (HS vs CF, 0.01 nM LXA_4_); ****p = 0.000000006 (CF + miR-181b inhibitor vs CF + miR-181b inhibitor + WRW4, 1 nM LXA_4_); ****p = 0.00000009 HS vs HS + WRW4, 1 nM LXA_4_).

To obtain further evidence of a “cause and effect” relationship between miR-181b and ALX/FPR2, we transfected CF-MΦs with a miR-181b inhibitor. Real-time PCR analysis showed that miR-181b levels were reduced by ~ 70% (p = 0.0013) in transfected cells. In these cells, ALX/FPR2 protein expression increased by ~ 40% (p = 0.0019) (Fig. 4A and B). Next, we determined whether miR-181b-mediated regulation of ALX/FPR2 had an impact on agonist-evoked biological responses. Because macrophage phagocytosis is the hallmark of resolution and it is strongly enhanced by pro-resolving lipid mediators, such as LX and Rv [13, 31], we examined phagocytosis of fluorescent-labeled zymosan particles as well as of the *Pseudomonas aeruginosa* strain PAO1 to mimic bacterial clearance from inflamed tissue. We exposed MΦs to increasing concentration of LXA_4_ (0.001–10 nM) and compared the phagocytic capability of healthy (HS) MΦs, CF-MΦs and CF-MΦs transfected with a miR-181b inhibitor. The zymosan phagocytic activity of unstimulated CF-MΦs was slightly, although not significantly, reduced compared to HS-MΦs (results not shown). When exposed to LXA_4_, HS-MΦs displayed a significant increment in zymosan and PAO1 uptake, which was maximal with 1 and 0.1 nM LXA_4_, respectively (Fig. 4C and D). In contrast, CF- MΦs showed a smaller increment in phagocytic activity when incubated with LXA_4_ (Fig. 4C and D). However, when these cells were transfected with the miR-181b inhibitor, an almost full recovery of LXA_4_- induced phagocytosis was observed, particularly at lower LXA_4_ concentrations (Fig. 4C and D). Receptor dependence was assessed using the WRW4 peptide, which abrogated LXA_4_-induced phagocytosis of zymosan, both in normal and CF cells (Supplementary Fig. 1). Under these experimental settings, we were unable to detect significant changes in the release of selected cytokines (IL-8, IL-10, IL-1β, RANTES, GM-CSF).

Collectively, these results demonstrate that changes in miR-181b expression impair the ALX/FPR2 proresolution signaling in CF cells.

## Discussion

Exuberant lung inflammation and infection, leading to respiratory insufficiency and death are trademarks of CF. Recent evidence indicates that in addition to a marked overproduction of pro-inflammatory mediators, an impairment in endogenous anti-inflammatory proresolving mechanisms does occur in CF^4,21^.

Here, we investigated on the ALX/FPR2 receptor, a GPCR that transduces signals by the endogenous proresolving mediators LXA_4_, RvD1 and ANXA1^12–14^ and it is regarded as a main proresolving receptor that intercepts multiple proresolution pathways. Since changes in ALX/FPR2 levels influence the outcome of an inflammatory response^32^ we evaluated ALX/FPR2 expression in two cellular models of CF, i.e. airway epithelial cells as a paradigma of the respiratory pathology and macrophages as key effectors of innate immune responses. In both models ALX/FPR2 levels were reduced compared to the normal counterpart (Figs 1 and 3). Combining this evidence with early data showing reduced LXA_4_ concentration in BAL from CF patients^21^; impaired LX biosynthesis, due to deficient 12-LO activity in CF platelets^4^; and lower ANXA1 in CF neutrophils^25^, we can conclude that the proresolution circuit converging on the ALX/FPR2 receptor is impaired in CF. Together with data showing beneficial actions of LXA_4_ in CF settings^33,34^ these results encourage further research to test whether the upregulation of the ALX/FPR2 pathway can be regarded as a suitable strategy to combat inflammation in CF patients. Along these lines, ALX/FPR2 and its proresolution agonists are defective in other human diseases characterized by non resolving inflammation^17–20^, confirming the relevant immunomodulatory role of this system.

The proresolving actions of LXA_4_ can be strengthened by the upregulation of ALX/FPR2 expression^27^. Therefore, acting on these mechanisms may help to develop innovative anti-inflammatory, proresolving pharmacology. We previously identified miR-181b as a main epigenetic regulator of ALX/FPR2 expression^26^. Here, we tested the hypothesis that the lower ALX/FPR2 expression in CF cells was related to changes in miR-181b level.

We present evidence of miR-181b upregulation in CF respiratory epithelial cells as well as in CF monocyte-derived macrophages (Figs 1 and 3). Although the role of this miR in inflammatory disorders remains to be conclusively defined, evidence indicates that it may exert pro-inflammatory functions, at least in selected settings. For instance, miR-181b regulates TNF-α-induced transcription of pro-inflammatory genes in liver cells^35^. It also regulates vascular inflammation mediated by NFkB^36^. More recently, a role of miR-181b in atherosclerosis and aneurysms has been proposed^37^. Our results with CF cells confirm that miR-181b levels can be elevated in pathological settings characterized by unresolved inflammation and provide clues to decipher the pathophysiological significance of this miR.

In the specific case of CF, a direct correlation between the genetic defect and miR-181b upregulation appears to occur. We in fact observed that miR-181b expression is under the control of CFTR. This was established in MΦs by experiments with CFTRinh-172, a selective CFTR inhibitor^38^ (Fig. 3), and in respiratory epithelium by using cells carrying the ΔF508 mutation and cells where the genetic defect was corrected by the overexpression of wild type CFTR (Fig. 3). Together, these data support the emerging concept of CFTR as epigenetic modulator^39^. Along these lines, it has been recently reported that CFTR regulates miR-125b expression with a bicarbonate-dependent mechanism^40^. Whether ion fluxes are also involved in CFTR-dependent miR-181b regulation remains to be determined.

On the other hand, our present results indicate that, at least in the CF model, the regulatory mechanisms of ALX/FPR2 expression may be cell specific. In fact, ALX/FPR2 mRNA was reduced in CF airway cells (Fig. 1), but not in CF-MΦs (data not shown), suggesting that transcriptional regulatory events are altered in CF respiratory epithelium. We previously documented the impact of epigenetic modifications of the ALX/FPR2 promoter and H3 histones on ALX/FPR2 expression^27,28^. Although the relevance of these changes remains to be determined in CF cells, our results clearly demonstrate that miR-181b controls ALX/FPR2 protein expression in both CF respiratory cells and MΦs (Figs 2 and 4). Moreover, in both cell types, by acting on miR-181b it was possible to enhance the functional responses of the pro-resolution agonist LXA_4_. In CF airway epithelial cells, we observed a significant increase in TEER (Fig. 2), an indirect readout of the monolayer integrity and of the strength of intercellular junctions, which are altered in CF cells and promote local inflammation^41^. In MΦs, a miR-181b inhibitor normalized LXA_4_-triggered phagocytic activity (Fig. 4). This was observed with both zymosan particles and *Pseudomonas aeruginosa*, which chronically colonizes the airways of the majority of CF patients and it is very difficult to eradicate. These results confirm that by blocking miR-181b it is possible to enhance LXA_4_ bioactions useful to control bacterial colonization in CF. On the other hand, under the present experimental settings, we were unable to detect significant changes in the release of selected cytokines by airway cells, in the presence or not of miR-181b inhibitor. This is likely to be related to the predominant antagonist action of LXA_4_ on the release of inflammatory cytokines, which can be uncovered by pre-exposing cells to pro-inflammatory stimuli. Along these lines, the downstream signaling leading to the enhanced LXA_4_-induced phagocytic activity, observed when miR-181 was inhibited, requires further investigation. Whether elements of the autophagy cascade are involved, as recently reported in murine and human macrophages, remains to be determined^31^. This aspect is particularly relevant in CF, since autophay activators can correct misfolded ∆F508 CFTR and promote clearance of Pseudomonas aeruginosa by ∆F508 CFTR macrophages^42–44^.

Regardless of these limitations, our present data indicate that the LXA_4_-ALX/FPR2 signaling may represent a novel target to stimulate the anti-inflammatory, anti-microbial defense in CF.

In conclusion, here we uncovered the upregulation of miR-181b in CF cells, which contributes to impair the endogenous anti-inflammatory, anti-microbial defense pathway centered on the ALX/FPR2 receptor. Together, these results expand our knowledge of mechanisms of CF inflammation and point to regulatory mechanisms of ALX/FPR2 expression as to potential targets for the development of innovative pharmacology for CF.

## Methods

LXA_4_ (5S,6R,15S-trihydroxy-7E,9E,11Z,13E-eicosatetraenoic acid) was purchased from Calbiochem, (Millipore, Billerica, MA), stored at −80 °C in ethanol and dissolved in the appropriate aqueous buffer immediately before use. The WRW4 peptide was purchased from Abcam (Cambridge, UK). CFTR inhibitor-172 was from Calbiochem. Growth media, fetal bovine serum (FBS), and supplements were from Gibco (Waltham, MA USA) unless otherwise indicated.

### Monocyte isolation and macrophage differentiation

Monocytes were isolated from peripheral blood (15 ml collected in sodium citrate-containing tubes) of healthy subjects [mean age 34 ± 4.6 (SD), 44% female] and age- and sex-matched CF patients [mean age 27 ± 5.4 (SD), 47% female] referring to the Regional Reference Center for Cystic Fibrosis, Atri (TE), Italy. Patients recruited for this study were free of pulmonary exacerbations at the time of recruitment and had not received i.v. antibiotics, nor steroids and non-steroidal anti-inflammatory drugs in the 2 weeks preceding blood withdrawal.

After dextran (6%) sedimentation, mononuclear cells were separated using Histopaque-1077 Ficoll (Sigma, Milan, Italy). Cells (12 × 10^6^) were suspended with serum-free RPMI medium and allowed to adhere to polystyrene plates for 1–2 h. Lymphocytes were aspirated and adherent monocytes were analyzed for purity by flow cytometry using an anti-CD-14 antibody (TÜK4 clone, Miltenyi Biotech, Calderara di Reno, Bologna, Italy). MΦ differentiation was obtained by exposing monocytes to RPMI supplemented with 10% FBS, 1% L –glutamine, 1% penicillin/streptomycin, and GM-CSF (10 ng/µl, Prospec, East Brunswick, NJ) for 7 days^13^. Purity of the isolated MΦ, as assessed by flow cytometric analysis of CD14 staining, was 100% (M.F.I. ratio = 7.6).

The study was approved by the Ethics Committee of the ASL Teramo, and carried out in accordance with the Declaration of Helsinki, as revised in 2004 and following the guidelines for observational studies published by AIFA (20.03.2008 GU n. 76 of 31.03.2008). Written informed consent was obtained by all human participants.

### Bronchial Epithelial Cells

Normal respiratory epithelial cells (16HBE14o-), CF airway cells homozygous for the ∆F508 mutation (CFBE41o-) and the genetically corrected CF airway cells CFBE41o- CEP-CFTR wt 6.2 kb (CFBE41o-/WT), all kindly provided by Dr. Dieter Gruenert, UCSF, USA, were seeded in fibronectin-coated plates and maintained in MEM medium supplemented with 10% FBS, 1% L-glutamine, 1% penicillin/streptomycin.

Primary human CF airway epithelial cells, isolated from different patients harboring the ∆F508 CFTR genotype subjected to lung transplantation, were obtained and provided by Dr. LJV Galietta (Telethon Institute of Genetics and Medicine, Pozzuoli, NA) as a public service of the Italian Cystic Fibrosis Research Foundation. Cells were grown in serum free growth medium (LHC9:RPMI 1640 1:1) supplemented with growth factors^45^. For differentiation, epithelia cells were cultured at high density (5 × 10^5^ cells/cm^2^) in air liquid interface condition on Transwell filters (0.4 µm pore Ø) for 8–10 days in differentiation medium (Ham’s F12, 2% FBS)^46^. Epithelium formation was confirmed by measuring TEER.

### Transfection of MΦs and CFBE41o- cells

Human MΦs were transfected with 10 nM miR-181b inhibitor (single-stranded modified RNA, miScript, Qiagen, Milan, Italy) or non-targeting single-strain RNA (Qiagen) using the INTERFERin reagent (Polyplus Transfection) as reported^26^. For CFBE41o- transfection, miR-181b inhibitor and negative control were diluted with 200 µl of Opti-MEM (Invitrogen, ThermoFisher Scientific, Waltham, MA USA) and combined with 4 µl of INTERFERin for 10 min at room temperature. This solution was immediately added to cells that were incubated at 37 °C.

### miR-181b analysis

miR-181b levels were determined as previously reported^26^. In brief, we used a silica-based spin column system (Norgen, Thorold, ON, Canada) for extraction of miRNA-enriched fractions. Samples were reverse-transcribed with the miScript II RT kit (Qiagen). Real-time PCR analyses were carried out with 1.5 ng of cDNA using specific primers (miScript Primer Assays) and a SYBR Green master mix (also from Qiagen) with a 7900HT Fast Thermal cycler (Invitrogen). miR-181b relative abundance was quantitated using the 2^−ΔΔCt^ method^47^. The exogenous cel-miR-39 or endogenous RNU6 and SNORD95 were used to normalize input cDNA.

### ALX/FPR2 expression

ALX/FPR2 mRNA was evaluated as in Pierdomenico *et al*.^26^. Total RNA was extracted using a silica-based spin column system (Norgen, Thorold, ON, Canada), quantified using a NanoDrop spectrophotometer (Thermo Scientific, Waltham, MA), and reverse-transcribed with the M-MLV Reverse Transcriptase (Sigma). Real-time PCR determinations were carried out using 500 ng of cDNA using the following primers: 5′-GGCCAAGACTTCCGAGAGAG-3′ (forward); 5′-CCGTGTCATTAGTTGGGGCT-3′ (reverse) and a SYBER ROX Real Master Mix (5 Prime, Hilden, Germany) with a 7900HT Fast Thermal cycler (Invitrogen). The GUSB gene was used to normalize cDNA input and ALX/FPR2 relative abundance was determined by the 2^−ΔΔCt^ method^47^.

ALX/FPR2 protein was assessed by flow cytometry. To this end, cells (1 × 10^6^ /sample) were incubated with 0.5 µg of the Human FPRL1/FPR2 APC-conjugated Antibody (R&D Systems, Minneapolis, MN, USA). Analyses were carried out using a FACS Canto flow cytometer equipped with the Diva software (BD Bioscience). Total ALX/FPR2 was measured in cells permeabilized with Permeabilizing Solution 2 (BD Bioscience). Membrane ALX/FPR2 was assessed using non permeabilized cells.

### MΦs phagocytosis

MΦs were transfected with 10 nM miR-181b inhibitor or non-targeting single-strain RNA vector using INTERFERin (Polyplus Transfection TM) and seeded in 24-well plates (2.5 − 5 × 10^5^ cells/well) 24 h post transfection. The following day, cells were washed twice with Dulbecco’s Phosphate-Buffered Saline (DBPS) and exposed to LXA_4_ or vehicle (0.01% EtOH) for 15 min at 37 °C. Fluorescein isothiocyanate (FITC)-labeled serum opsonized zymosan (Zym) A (from Saccharomyces cerevisiae) particles (15 ng/well) were added to cells for 30 min at 37 °C. MΦs were washed twice with DPBS and added of 100 µl of trypan blue (0.03% in DPBS) to quench extracellular fluorescence. Phagocytosis was assessed by measuring fluorescence with a Synergy H1 microplate reader (Biotek, Milan, Italy).

For phagocytosis of *Pseudomonas aeruginosa*, the PA01 strain, constitutively expressing the green fluorescent protein (GFP), kindly provided by Dr. Gerald Pier (Department of Medicine, Brigham and Women’s Hospital, Harvard Medical School, Boston, MA) upon material transfer agreement, was grown to sub-confluence in 20 ml of tryptic soy broth until reaching an optical density (OD_600_) = 0.45 ± 0.05, corresponding to ~ 2 × 10^8^ colony forming units/ml. After centrifugation (2,700 rcf, 15 min, 4 °C) and addition of 1 ml of Ca/Mg-free PBS, PA01-GFP (10 µl) was added to monocyte-derived MΦs (2 × 10^5^ cell/well) in 24 well plates at a 1:200 cell:bacteria ratio. After 1 h at 37 °C in a humidified 5% CO_2_ incubator, the excess of bacteria was aspirated, plates were washed, and extracellular PA01-GFP was quenched with trypan blue (0.02% w/vol, 200 µL/well, ~ 1 min). Phagocytosis of PA01-GFP was determined by measuring total fluorescence (Ex 485 nm/Abs 530 nm) using a plate reader (Synergy, BioTek).

### Trans epithelial electrical resistance (TEER)

CFBE41o- cells, transfected with 10 nM miR-181b inhibitor or non-targeting negative control single-strain RNA vector using INTERFERin (Polyplus Transfection TM) were seeded (6 × 10^5^ cells/well) in Transwell permeable supports (6.5 mm insert, 24 well plate) (Corning, NY, USA) 24 h post transfection. The following day, cells were washed twice with DPBS and treated with LXA_4,_ LXA_4_ + WRW4 or vehicle (0.01% EtOH) in DPBS. After 4 h, TEER was measured by a EVOM Voltmeter (World Precision Instruments, Sarasota, FL, USA).

### ALX/FPR2 promoter activity

CFBE41o- and 16HBE14o- cells were seeded in 6-well plates. After 24 h, cells were washed twice with PBS and supplemented with DMEM medium containing 1% FBS. Cells were then transfected with 4 μg of the pGL4 basic plasmid or pGL4 basic + the 346 bp sequence of the ALX/FPR2 core promoter as reported^28^. Luciferase activity was measured 48 h post-transfection using the luciferase assay kit (Promega) according to the manufacturer’s protocol. Values were normalized for protein concentration as reported^28^.

### Statistical analysis

Results are reported as mean ± SEM. Statistical significance was evaluated by the Student’s T-test. P values < 0.05 were taken as statistically significant.

## Electronic supplementary material

Supplementary Information
