# Supplementary material for: microRNA-181b is increased in cystic fibrosis cells and impairs lipoxin A4 receptor-dependent mechanisms of inflammation resolution and antimicrobial defense
Source: Sci Rep. 2017 Oct 18;7:13519. doi: 10.1038/s41598-017-14055-y (PMC5647414; doi:10.1038/s41598-017-14055-y)
Supplement: Supplementary file 1 — Supplementary Information [file 41598_2017_14055_MOESM1_ESM.pdf]

microRNA-181b is increased in cystic fibrosis cells and impairs lipoxin A_4 receptor-dependent mechanisms of inflammation resolution and antimicrobial defense

Anna Maria Pierdomenico[1,3], Sara Patruno[2,3], Marilina Codagnone [2,3], Felice Simiele[2,3], Veronica Cecilia Mari[2,3], Roberto Plebani[2,3], Antonio Recchiuti[2,3] and Mario Romano[2,3]

[1]Department of Medicine and Aging Sciences; [2]Department of Medical, Oral, and Technological Sciences; [3]Center on Aging Science and Translational Medicine (CeSI-MeT) "G. D'Annunzio" University of Chieti-Pescara, 66013 Chieti, Italy.

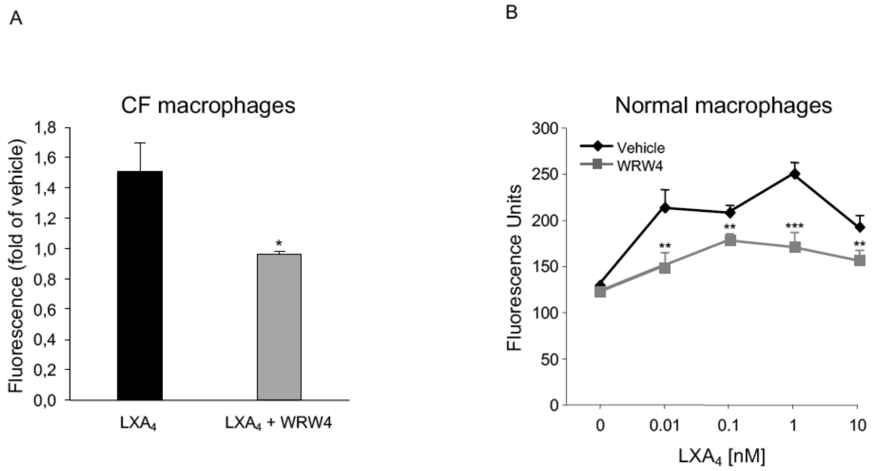

Supplementary Figure 1

Legend

Supplementary Figure 1. LXA_4-induced phagocytosis is abrogated by WRW4. (**a**) MΦs (2.5 x 10^5/well) from 3 CF patients were exposed to 0.1 nM LXA_4 in the presence or not of WRW4 (10 μM). Phagocytosis of FITC-labelled zymosan particles was assessed as indicated in the legend to Fig. 4. Data are expressed as fold of phagocytosis measured in cells exposed to vehicle. Bars depict mean ± SEM. *p = 0.05. (**b**) MΦs (2.5 x 10^5/well) from HS were exposed to the indicated LXA_4 concentration, alone or in the presence of WRW4 (10 μM). Phagocytosis of FITC-labelled zymosan particles was assessed as above. Data are mean ± SEM from experiments with cells from 3 healthy donors. **p = 0.009 (0.01 and 10 nM LXA_4); **p = 0.003 (0.1 nM LXA_4); ***p = 0.0007 (1 nM LXA_4) vs vehicle.
